# Directional gene expression and antisense transcripts in sexual and asexual stages of *Plasmodium falciparum*

**DOI:** 10.1186/1471-2164-12-587

**Published:** 2011-11-30

**Authors:** María J López-Barragán, Jacob Lemieux, Mariam Quiñones, Kim C Williamson, Alvaro Molina-Cruz, Kairong Cui, Carolina Barillas-Mury, Keji Zhao, Xin-zhuan Su

**Affiliations:** 1 Laboratory of Malaria and Vector Research, National Institute of Allergy and Infectious Diseases, National Institutes of Health, 9000 Rockville Pike, Bethesda, Maryland 20892, USA; 2 Bioinformatics and Computational Biosciences Branch, National Institute of Allergy and Infectious Diseases, National Institutes of Health, 9000 Rockville Pike, Bethesda, Maryland 20892, USA; 3 Department of Biology, Loyola University Chicago, Chicago, Illinois 60660, USA; 4 Systems Biology Center, National Heart, Lung, and Blood Institute, National Institutes of Health, 9000 Rockville Pike, Bethesda, Maryland 20892, USA

## Abstract

**Background:**

It has been shown that nearly a quarter of the initial predicted gene models in the *Plasmodium falciparum *genome contain errors. Although there have been efforts to obtain complete cDNA sequences to correct the errors, the coverage of cDNA sequences on the predicted genes is still incomplete, and many gene models for those expressed in sexual or mosquito stages have not been validated. Antisense transcripts have widely been reported in *P. falciparum*; however, the extent and pattern of antisense transcripts in different developmental stages remain largely unknown.

**Results:**

We have sequenced seven bidirectional libraries from ring, early and late trophozoite, schizont, gametocyte II, gametocyte V, and ookinete, and four strand-specific libraries from late trophozoite, schizont, gametocyte II, and gametocyte V of the 3D7 parasites. Alignment of the cDNA sequences to the 3D7 reference genome revealed stage-specific antisense transcripts and novel intron-exon splicing junctions. Sequencing of strand-specific cDNA libraries suggested that more genes are expressed in one direction in gametocyte than in schizont. Alternatively spliced genes, antisense transcripts, and stage-specific expressed genes were also characterized.

**Conclusions:**

It is necessary to continue to sequence cDNA from different developmental stages, particularly those of non-erythrocytic stages. The presence of antisense transcripts in some gametocyte and ookinete genes suggests that these antisense RNA may play an important role in gene expression regulation and parasite development. Future gene expression studies should make use of directional cDNA libraries. Antisense transcripts may partly explain the observed discrepancy between levels of mRNA and protein expression.

## Background

The malaria parasite *Plasmodium falciparum *remains a major causative agent of human disease, killing ~800,000 people each year [1]. To facilitate development of drugs and vaccines to control malaria and to better understand the biology of the parasite, the genome of the 3D7 strain was sequenced and published in 2002 [2], which constitutes a significant achievement in malaria research. Like most genome projects of other species, the initial gene prediction and annotation were largely accomplished using *in silico *prediction, which may lead to errors in some gene models. Indeed, up to 30% of the detected transcripts were found to be unannotated, even in the well characterized *Drosophila *genome in a high-density microarray analysis [3]. Similarly, approximately a quarter of the predicted gene models of the *P. falciparum *were found to contain errors in a study of cDNA sequence analysis [4].

Large numbers of *P. falciparum *expressed sequence tags (ESTs) or cDNA sequences [4-9], and structural/regulatory RNA [10-12] have been reported from *P. falciparum*; however, efforts to sequence and obtain full-length cDNA have been hindered by the AT-rich DNA sequences in the genome that has an average of ~80% A+T [2]. First, the traditional Sanger sequencing approach generally requires cloning cDNA sequences into bacteria, and many of the *P. falciparum *DNA sequences are unstable in bacteria and are often deleted during the cloning process. Second, a full-length cDNA will contain 5' and 3' untranslated regions (UTRs). The 5' and 3' UTRs of the parasite--including the polyA tail--are usually very AT rich and are difficult to sequence. These difficulties have prevented the cloning and sequencing of many *P. falciparum *cDNA sequences, particularly transcripts larger than 2 kb. Recently, many next-generation sequencing platforms have been introduced for transcriptome and genome analyses [13-16]. These methods are low-cost, high-throughput, and do not require cloning DNA into bacteria. Indeed, several recent studies have reported large-scale sequencing of transcriptome and epigenome from *P. falciparum*, including samples from clinical isolates [17-20]. These studies identified large numbers of additional intron-exon splicing junctions missed by the initial genome annotation, alternative splicing events, and antisense transcripts, and have greatly improved EST coverage and genome annotation; however, these studies primarily focused on genes expressed in asexual stages. No systematic cDNA sequence analysis or verification of gene models has been done for gametocyte (G) and mosquito stages, which are expected to have many stage-specific expressed genes.

Here we report results of Illumina-based sequencing of mRNA from two gametocyte stages (GII and GV), ookinete (Oo), and four time points of erythrocytic stages representing ring (R), early trophozoite (ET), late trophozoite (LT), and schizont (Sc). We also sequenced strand-specific (unidirectional) cDNA libraries from ET, Sc, GII, and GV to systematically investigate antisense transcripts. After comparing our RNA sequences with the latest gene models incorporating updates described in the recent reports [17-19], we detected more than one thousand additional errors in gene models, alternatively spliced events including stage-specific alternatively spliced genes, and antisense transcripts in G and Oo stages. Our data suggest that antisense RNA plays a role in gene expression regulation in the sexual stages.

## Results

### RNA-seq from *P. falciparum *developmental stages

To investigate changes in *P. falciparum *gene expression and regulation during its life cycle, we initially constructed seven Illumina RNA-seq single-end libraries (bidirectional) from the 3D7 parasite (Table 1). Polyadenylated mRNA was affinity purified using oligo(dT) beads and converted to cDNA using random hexamers. A total of 127 million 36-bp reads (51-bp for the Oo library), ranging from 9.2 to 55.1 million per library, was first obtained (Table 1). The sequence reads were aligned to the 3D7 reference genome (PlasmoDB version 7.1) using TopHat, a Bowtie-based software capable of aligning split reads [21]. Except for the Oo library, for which one mismatch was allowed due to the longer reads, only those reads with a unique alignment and zero mismatch to the reference genome were used in further analysis. Approximately 35% of the reads were successfully mapped to the reference genome. Although the Oo stage generated the largest number of raw reads (55,140,294), only 714,769 reads (1.3%) were mapped to the 3D7 reference genome due to large amount of *Anopheles gambiae *RNA.

**Table 1:** Summary of sequence reads obtained from different RNA-Seq libraries

Library	Total reads	Mapped reads	Unmapped reads	% mapped reads
R_BD^1^	13,456,136	4,557,242	8,898,894	33,86%
ET_ BD ^1^	14,505,855	5,801,368	8,704,487	39,99%
LT_ BD ^1^	14,599,502	7,567,175	7,032,327	51,83%
Sc_ BD ^1^	9,861,818	1,487,604	8,374,214	15,08%
GII_ BD ^1^	9,294,474	3,647,906	5,646,568	39.25%
GV_ BD ^1^	10,641,921	6,610,820	4,031,101	62.12%
Oo_ BD ^2^	55,140,294	714,769	54,425,525	1.3%
LT_SS^3+^	13,928,058	8,852,802	5,075,256	63.56%
Sc_ SS ^3+^	14,360,448	8,386,042	5,974,406	58.40%
GII_ SS^3+^	9,331,268	4,952,677	4,378,591	53.08%
GV_ SS^3+^	20,197,230	6,534,612	13,662,618	32.35%

R, ET, LT, Sc, GII, GV, and Oo correspond to ring, early trophozoite, late trophozoite, schizont, gametocyte II, gametocyte V, and ookinete stages, respectively. BD represents bidirectional libraries, and SS represents strand-specific libraries. "Total reads" corresponds to the initial output of sequencing after analysis with Illumina pipeline v3.1; "mapped reads" refers to numbers of reads mapped to the Pf3D7 reference genome (PlasmoDB version 7.1) allowing one single hit and no mismatches ^(1)^, one mismatch ^(2)^, or up to two mismatches ^(3) ^at the reads. ^+ ^indicates the total numbers of reads obtained after de-multiplexing.

### Antisense transcripts and strand-specific cDNA libraries

Alignment of the reads from the bidirectional libraries to the 3D7 reference genome revealed 363 splicing events characterized by 5'CU-AC3' junctions that matched the reverse and complement sequence of the canonical intron-exon boundary of 5'GT-AG3', suggesting intron-exon junctions in antisense. These antisense junctions affected a total of 246 genes, 56 of which were also described in a recent study [18] (Additional file 1). Interestingly, 79% of the genes displaying antisense junctions were multiple-exon genes, in agreement with the previously mentioned study where up to 86% of the antisense junctions detected were mapped to intron-containing genes [18]. Furthermore, 55% of the antisense junctions identified in this study specifically overlapped with intron-exon boundaries in the sense direction of transcription. The majority (95.6%) of these putative antisense transcripts were detected in G and Oo stages, particularly at GV (51.8%), suggesting that these antisense transcripts may play an important role in gene expression in G and Oo development such as suppressing the expression of genes essential for asexual growth and replication.

**Figure 1:**
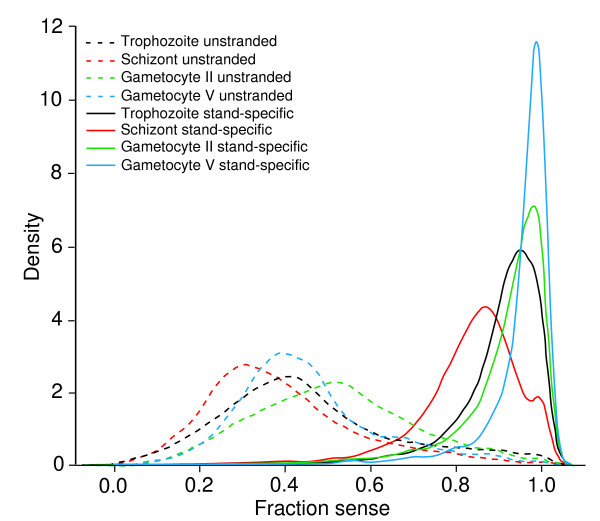
**Density distribution of reads from bidirectional and strand-specific libraries of late trophozoite, schizont, gametocyte II, and gametocyte V**. The sense fraction was calculated based on the ratio between sense and total number of reads present at each exon in the genome (total of 14,777 exons). Density represents the number of genes that have a particular ratio of forward reads over total number of reads for that gene. Strand-specific libraries (solid lines) show directionality characterized by high frequencies of exons with fraction values close to 1, whereas those from bidirectional libraries (dashed lines) have the majority of exons with sense fractions between 0.2 and 0.5.

**Figure 2:**
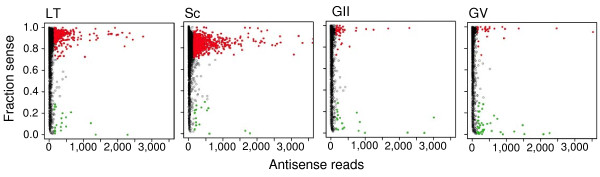
**Scatter plots showing exons with various numbers of antisense reads at different fractions of sense reads from the strand-specific libraries**. The fraction of sense reads was plotted against the number of antisense reads from late trophozoites (LT), schizonts (Sc), gametocyte stage II (GII), and gametocyte stage V (GV) for each of the 14,777 exons in the genome. Green dots represent exons with a high number of antisense reads (> 150) and low level of sense fraction (< 0.3), and reds dots are those with high levels of sense fraction (> 0.7) and antisense reads (> 150 reads).

To further investigate the scale and pattern of antisense transcription, we obtained reads from strand-specific (unidirectional) cDNA libraries of four different stages of *P. falciparum *and mapped the sequenced read pairs to the parasite reference genome. We obtained 13.9, 14.3, 9.3, and 20.2 million of bar coded 45-bp (after removing 6-bp barcode) paired-end reads from LT, Sc, GII, and GV stages, respectively (Table 1). We first evaluated the quality of our strand-specific libraries by plotting the fraction of sense reads in each exon in the genome. As expected, the vast majority of exons was transcribed overwhelmingly in the sense direction, with a fraction of sense reads approaching 1; in contrast, distribution of sense fraction was centered around 0.4 for the bidirectional libraries (Figure 1). We noted that the distribution of sense ratios was different for individual stages, with GII and GV having distributions of sense ratio characterized by smaller variance than LT and Sc (Figure 1). While we cannot exclude the possibility that this is due to technical variability in the efficiency of second-strand digestion during the library preparations, it is possible that there is a tighter directional control of gene expression in G along with more bidirectional promoter activity during the Sc stage. Interestingly, the majority of genes with large numbers of antisense reads either had low fractions of sense transcripts (possibly suppressed by antisense) or had relatively high levels of sense transcripts (bidirectional transcription) (Figure 2), although again we cannot rule out that some of the antisense transcripts were due to background noises from reverse transcription.

### Stage-specific antisense transcripts

**Figure 3:**
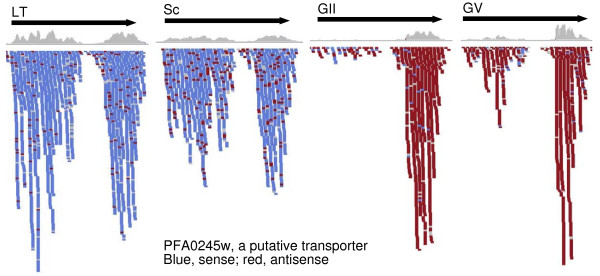
**Change in transcript direction in gene PFA0245w, a putative transporter, during development from asexual to sexual stages**. LT, late trophozoite; Sc, schizont; GII, gametocyte stage II; GV, gametocyte stage V. The black arrow bars indicate open reading frame direction. The blue color indicates reads in sense direction, and those in red are reads in antisense direction. Note that there are more red reads in Sc than in LT, and at the 5' end in the GV than in GII. The gap without coverage represents a repetitive region where reads were removed.

To account for differences in each library, we modeled each exon with n sense reads as a sample from a binomial distribution, B(n, r), with n successes each with probability r, where r is estimated individually for each library from the overall ratio of sense to antisense (S:AS). We then used -log(p), where p is calculated from a binomial test, as a measure of antisense transcription that could be applied to all exons. We identified 343 exons that had large numbers of antisense reads (-log(p) ≥ 150) in one or more of the four stages analyzed (Additional file 2). Again the GV stage had the largest number of genes covered with dominant antisense transcripts (176), including 40 that had strong antisense transcription accompanied by an almost total depletion of sense transcripts (Additional file 2 and Figure 2). These 343 exons belonged to 312 genes, 70 of which are also detected as antisense-containing genes after the identification of CU-AC junctions in our bidirectional libraries (Additional file 1). There were also genes with high levels of antisense reads at GV but high levels of sense transcript in LT and Sc, and the two transcripts are expressed reciprocally in a dynamic transition process as the parasite grows from LT to Sc, GII, and finally to dominant antisense transcripts in GV (Figure 3 and Additional file 2). These could represent genes that are expressed at high levels in asexual stages and become silent in expression at G stages when antisense transcriptions increase.

### Physical locations of antisense transcripts

**Figure 4:**
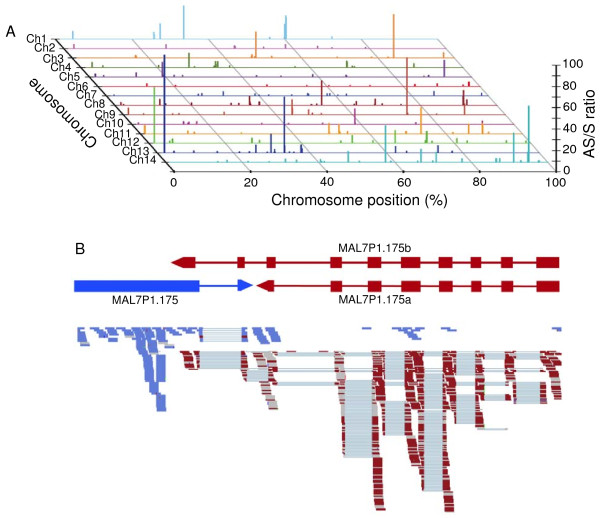
**Distribution of antisense:sense ratio (AS:S) across the 14 parasite chromosomes and an example of 'run-through' transcript in tail-to-tail orientation**. (A) Distribution of antisense:sense (AS:S) ratios across the 14 chromosomes (Ch1-14) in the GV stage. (B) A pair of genes showing transcripts possibly 'running through' into a neighboring gene in opposite orientation, possibly suppressing the expression of the neighboring gene. MAL7P1.175b is not annotated in the current *Plasmodium falciparum *genome.

It has been shown that antisense-sense ratio (AS:S) was unevenly distributed across the mouse genome, with some chromosomes having higher (or lower) AS:S pair density than the genome average [22]. Exons with high AS/S in the *P. falciparum *genome were randomly distributed across the 14 parasite chromosomes (Figure 4A). Visual inspection of individual genes revealed an uneven distribution of antisense reads within some genes. In a number of cases, antisense reads were markedly clustered at the 3' end of the exon of predicted gene models (Figure 3), suggesting potential bidirectional promoter activity [23]. Run-over transcriptions into neighboring genes appeared to occur in genes arranged in tail-to-tail orientation (Figure 4B), which may prevent the neighboring genes from being transcribed in the sense direction.

### Functional categories of genes with antisense transcripts

The majority of the 312 genes with high levels of antisense transcripts encoded conserved *Plasmodium *protein with unknown function or were small transcripts (16 RNAiz or "exons") that could represent regulating RNA or could have been previously annotated with a random directionality (Additional file 2). Among the genes with predicted functions, 22 were associated with transmembrane transport and 16 were involved in protein/intracellular transport (Additional file 2). Additionally, 16 genes encoded ribosomal proteins including 5 organelle ribosomal proteins, 3 genes encoded zinc finger proteins, and 2 encoded histone proteins. Gene ontology enrichment analysis on molecular function showed significant enrichment in cell adhesion, protein binding, and transport activities, and cellular component clustering identified significant enrichment of ribosomal subunits (Additional file 3).

### Detection and confirmation of intron-exon junctions

**Figure 5:**
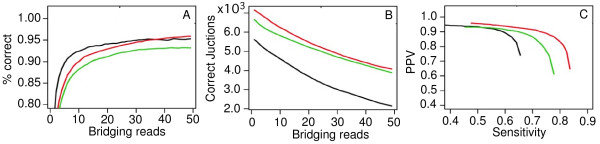
**Comparison of RNA-seq reads in matching intron-exon junctions of predicted gene models**. (A) Relationship of the numbers of reads bridging an intron and the chance of the intron matching one intron in the predicted gene models. (B) The numbers of introns matching those in the predicted gene models decrease as the numbers of reads bridging an intron increase. (C) Plot of sensitivity against positive predictive value (PPV). Sensitivity is defined as the numbers of matched intron-exon junctions between the predicted gene models and RNA-seq/numbers of intron-exon junctions from predicted gene models, and PPV is the numbers of matched intron-exon junctions between predicted gene models and RNA-seq/total numbers of intron-exon junctions identified from RNA-seq. Red lines, from our strand-specific libraries; green, data from Bartfai et al. [19]; black, data from Otto et al. [17].

Due to differences in read length and nature between our strand-specific and bidirectional libraries, we analyzed the two datasets independently. We first aligned the sequence reads from the strand-specific libraries to the genome sequence using TopHat, which outputs putative junctions in a gene model-independent approach. We then evaluated the performance of individual libraries and parameter settings using the annotated junctions as a validation set. Based on the number of currently annotated junctions identified and the number of mismatched junctions, we estimated the rate of false positives and false negatives at different parameter settings. As expected, the percentage of intron-exon matches increased along with the increase of numbers of bridging reads, reaching ~92% matching junctions at 15-read coverage and leveling off at ~95% matching with 40-read coverage (Figure 5A). We also compared our data with those from two recent RNA-seq studies in intron-exon matching [17,19]. Both our data and those from Otto et al. [17] reached a plateau of ~95-96% matches when the junctions were covered with 30 or more reads. Our four strand-specific libraries matched 7,152 out of the total of 8,553 predicted junctions (83.6%) at PlasmoDB if only one bridging read was considered, and 6,240 junctions (73.0%) if 10 bridging reads were used (Figure 5B and Additional file 4). Our sequences matched slightly more predicted introns than those of the two previous studies (Figure 5B), which can partly be attributed to the inclusion of sexual stages in our dataset. Our strand-specific RNA-seq data had slightly greater positive predictive value (PPV, defined as the numbers of matched intron-exon junctions between predicted gene models and RNA-seq/total numbers of intron-exon junctions identified from RNA-seq) at a given sensitivity (defined as the numbers of matched intron-exon junctions between the predicted gene models in PlasmoDB v7.1 and RNA-seq/numbers of intron-exon junctions from predicted gene models), which we again attribute to the additional lifecycle stages represented in our libraries (Figure 5C). For example, at PPV = 0.9, ~75% of intron-exon junctions from our RNA-seq matched those in PlasmoDB, whereas at the same PPV, ~65% intron-exon junctions from Bartfai et al. RNA-seq and ~55% from Otto et al., RNA-seq matched the intron-exon junctions in PlasmoDB (Figure 5C).

Using the RNA-seq data from our strand-specific libraries as well as those from the two previous studies and a PPV = 0.9 cutoff, we identified 1,202 intron-exon junctions not present in PlasmoDB v7.1 (Additional file 5). Using the same PPV = 0.9 cutoff, our RNA-seq alone detected 692 novel junctions. As expected, the datasets from Otto et al. and Bartfai et al. shared more junctions because their data did not include sequences from sexual stages.

We also identified 1,028 new junctions using the bidirectional libraries (Additional file 1). Among the 1,028 junctions, 667 were found uniquely at one stage, and the majority (93.5%) of the stage-specific junctions were from G or Oo stages, with 433 from GV, 100 from GII, and 91 from Oo.

### Correlation of gene expression of various datasets

**Figure 6:**
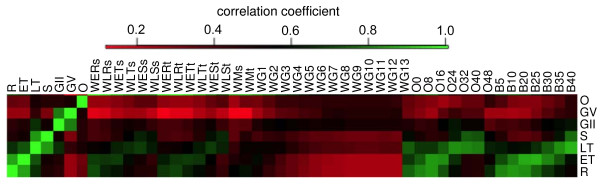
**Comparison of expression patterns between our data and those from previous studies**. Samples starting with "W" are from Le Roch et al. [24,25]. Samples starting with "O" are from Otto et al. [17], and the numbers are hours when RNA samples were isolated after erythrocyte invasion. Those with "B" are from Bartfai et al. [19], and again the numbers are hours after invasion when the RNAs were isolated. Green indicates positive correlation, and red suggests negative correlation. R, ring; ER, early ring; LR, late ring; ET, early trophozoite; LT, late trophozoite; Sc, schizont; ESc, early schizont, LSc, late schizont; M, merozoite; GII, gametocyte II; GV, gametocyte V; G1-G13, day 1 to day 13 gametocyte samples.

To compare the observed expression between our RNA-seq data and those published data from RNA-seq and microarray analysis, we calculated the Pearson correlation coefficient using the expression values at the seven time points under this study with data from four previous studies [17,19,24,25] (Figure 6). The expression levels estimated from our asexual stages correlated well with those of previous studies; however, correlation for the G stages was relatively low compared with the microarray data from Young et al. [25]. Generally, our data correlated better with data from similar RNA-seq than those from microarray (Figure 6).

### Stage-specific expressed and/or spliced genes

The malaria parasite has a complex life cycle with different morphology and functional characteristics associated with specific gene expression patterns [24,26]. Sequencing cDNA from multiple stages of parasite development, particularly the Oo, allowed us to catalog stage-specific expressed genes. We compared the fold changes in read coverage between ring (R) and the other six stages. Stage-specific genes or genes showing significant changes in expression were identified (Additional file 6). A total of 1,129 genes were found to change significantly in expression in one or more stages compared with those of R. In particular, there were 57 genes that were expressed significantly higher (35) or lower (22) in Oo than those in R stage. These genes included secreted Oo adhesive protein, TRAP-related protein, Oo capsule protein, and secreted Oo protein that are associated with development in mosquito midgut. There were also a large number of genes (282) that are expressed at higher levels only in GV stage, the majority of which are *Plasmodium *conserved proteins, reflecting a large number of uncharacterized G-specific genes. This analysis was performed exclusively using data from the bidirectional libraries, where Oo data were available, and some signals could come from antisense transcripts.

### Alternative spliced genes

**Figure 7:**
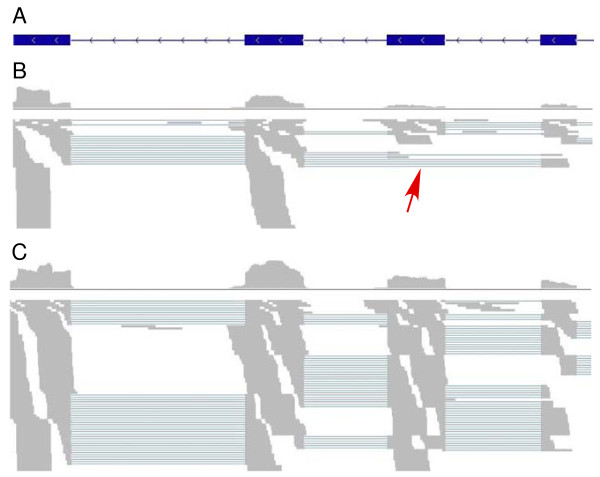
**An example of stage-specific alternatively spliced genes (exon skipping) in two different developmental stages**. (A) The predicted gene model of MAL13P1_42 (only part of the gene is shown); (B) reads from the bidirectional ring library; (C) reads from bidirectional late trophozoite library. The red arrowhead points to reads with a skipped exon.

Another mechanism of gene expression regulation is alternative splicing, which has been reported in *P. falciparum *previously [4,17,18]. Using the bidirectional libraries, we detected 201 new alternatively spliced events affecting 178 genes (Additional file 1), including 124 alternative splicing isoforms that were only found in one stage (stage specific) (Figure 7 and Additional file 1). Even though we cannot eliminate the possibility that some isoforms were expressed in undetectable levels at a particular stage, the stage-specific isoforms identified in this study could suggest a special role for these genes in parasite differentiation and development.

### Experimental verification of new and alternatively spliced isoforms

To validate the intron-exon junctions and alternatively spliced events detected by our RNA-seq, we randomly selected 63 splicing junctions identified from the bidirectional libraries and 55 junctions from the strand-specific libraries (Additional file 7). We designed primers flanking the newly described splice sites and PCR amplified cDNA from the same RNA samples used for library construction. Of the 63 junctions from the bidirectional libraries, 41 (~70%) were confirmed by PCR. Among the 22 that were found to be incorrect, 17 (77%) were located in areas of repeats in the genome. Based on these results, we filtered out all the detected junctions partially overlapping with repeat regions and/or without GT-AG site (as shown in Additional file 1), leading to an estimated confirmation rate of ~89% (41/46).

From the 55 junctions randomly selected from the strand-specific libraries, 45 were experimentally validated and 10 were demonstrated to be wrong, presenting a validation rate of 81.8% (Additional file 7). Again, after removing one in repetitive regions, the validation rate increased to 83.6%.

## Discussion

Several recent studies have reported large numbers of cDNA sequences that have greatly improved genome annotation and gene models in the *P. falciparum *genome [17-19]; however, none of the studies presented sequences from G or stages from mosquito. In this study, we obtained cDNA sequences from GII, GV, Oo, and four time points from asexual stages as well as strand-specific cDNA sequences from LT, Sc, GII and GV. We identified many unknown splicing junctions and stage specific expressed genes, including Oo-specific genes. Alignment of our sequences to the 3D7 genome sequence produced good matches of intron-exon junctions between our sequences and the gene models in the genome (> 6,000 of the 8,553 predicted junctions with 10 or more reads). We showed that Gs appear to have sense transcripts characterized by smaller variance of antisense than the asexual stages.

Natural antisense transcripts (NATs) have been widely recognized as an important mechanism of post-transcriptional regulation in both prokaryote and eukaryotic organisms [27-29]. In the human and mouse genomes, up to 72% of all genomic loci are found to have both sense and antisense transcripts [22,29]. Antisense transcripts have been shown to play a role in sense RNA transcription, pre-mRNA splicing, RNA editing, stability, and transport, and in regulation of translation [27]. NAT can regulate gene expression through several mechanisms [30]. In the transcriptional interference model, two bulky RNA polymerase II complexes on opposite DNA strands may interfere with one another, arresting transcription in one direction. In the RNA masking model, an antisense may mask a splice site on the sense pre-mRNA sequence, leading to an alternative splicing event. Formation of double-stranded RNA such as RNA editing and RNA interference is another type of regulatory mechanism, which may lead to degradation of sense transcripts (RNAi). In chromatin remodeling mechanism, transcription of non-coding antisense transcripts may be also involved in monoallelic gene expression such as genomic imprinting, X-inactivation and clonal expression of lymphocyte genes. Antisense transcripts can silence the expression of nearby genes through chromatin remodeling, most likely through the recruitment of histone-modifying enzymes [30]. NATs have been reported from *P. falciparum *previously [4,18,31,32], and antisense RNA or oligodeoxynucleotides have also been used to regulate gene expression in the parasite [33-35]. Our observation that the majority of the genes in schizont are expressed with S/AS pairs (Figure 1 and 2) is consistent with these previous reports and with the observations in human and mouse in which the majority of genes are expressed in both directions [22,29]. Although we do not have additional experimental evidence to confirm that the antisense transcripts we found here play a role in gene expression regulation through a specific mechanism, the presence of high levels of antisense RNA in a stage specific manner suggests that these antisense transcripts are likely associated with some unique features of the parasite developmental stages. Although the mechanism of RNAi has been shown to be absent in malaria parasites [36], pairing of antisense and sense transcripts may still play a role in gene expression through non-RNAi mechanisms. The mapping of ~86% of all antisense reads to intron containing genes suggests that antisense may play a role in intron splicing, possibly through the mechanism of masking the sense splicing sites; the predominant presence of antisense transcripts in intron-containing genes could be due to promoter activities of the introns. Of course, some of the genes with antisense coverage could also be artifacts produced by DNA-dependent DNA polymerase activity of reverse transcriptase [37]; however, it is difficult to imagine that one artifact only occurred in one stage such as G but not in other stages, because the samples were processed similarly at the same time.

An interesting observation from our strand-specific library sequences was the changes in the numbers of genes expressed in either a single direction (sense or antisense only) or in different S/AS mixtures in different stages. Whereas the majority of the genes in schizont are transcribed in mixtures with 10-30% RNA in the opposite directions of the major transcripts (Figure 1 and 2), the sexual stages appear to have a higher proportion of genes transcribed in higher S/AS ratios, suggesting a gradual shifting from more genes with mixture of transcripts in both directions in early G to more single-direction transcripts in mature G. Similarly, there appeared to be more genes with strand-specific transcripts in LT than Sc stage. Interestingly, it has been shown that the mouse X chromosome contains fewer bidirectional pairs of S/AS transcripts than the autosomes, and S:AS pairing is also associated with imprinted loci [22]. High levels of strand-specific transcripts in G stages will lead to fewer S/AS pairs in these stages, suggesting the possibility of a similar mechanism in regulating sexual development in the malaria parasite mediated through antisense RNA. Further investigation on the patterns of transcription direction in more stages will provide additional information on the changes in S:AS ratios and the relationship of S:AS ratio variation and parasite development cycle. The observation may also help explain the lack of correlation in expression level of RNA transcript and protein [38,39] and suggests that analysis of RNA expression should be conducted using strand-specific cDNA libraries so that more precise transcriptional patterns can be characterized.

The mature gametocyte (GV) is a unique sexual stage that is developmentally arrested but can quickly resume development, producing male and female gametes as soon as it is taken into a mosquito midgut. In the rodent malaria parasite *Plasmodium berghei*, it has been shown that many genes are transcribed but not translated in the G, which is regulated by a mechanism--termed "translational repression" mediated by DDX6-class RNA helicase, DOZI (development of zygote inhibited)--found in a complex with mRNA species in cytoplasmic bodies [40-42]. Our data showing the presence of large numbers of antisense transcripts (Additional file 1 and Additional file 2) in G suggest that these antisense transcripts could also be involved in gene expression regulation, either being part of the described translational suppression complex or other unknown mechanisms.

Using strand-specific reads, we were able to confirm ~84% and 73% of the intron-exon junctions in the predicted gene models if 1 or ≥ 10 bridging reads were used, respectively. Compared with the sequences from two previous reports [17,19], our strand-specific sequences appeared to match the predicted intron-exon junctions better (higher percentage) than those of the other two studies. The performance of a set of libraries in identifying splice junctions depends on the read lengths, base quality, total number of reads, the fraction of the total number of transcripts expressed at appreciable levels, and sequence alignment parameters. One explanation for the observed higher matches from our data is likely that our libraries included transcripts from G and Oo, whereas the other two studies had sequences from asexual stages only and therefore would miss the splice junctions in sexual stages. Our sequences also identified nearly 700 putative intron-exon junctions using a PPV = 0.9 cutoff. As expected, many of the new junctions were from genes that were expressed in Gs and were not characterized in previous studies. Another potential explanation for lower junction coverage for the data from Otto et al. could be related to bias amplification using high PCR extension temperature. We have shown that using an extension temperature of 60°C can increase the coverage of sequences in non-coding regions [43]. Intron-exon junctions at 5' or 3' UTR may not amplify well using standard PCR conditions that were likely employed by Otto et al. [17]. We used 60°C extension temperature, and Bartfai et al. [19] used a linear amplification method that avoided biased sequencing of the AT-rich *Plasmodium *genome; these two approaches therefore produced similar numbers of junction calls (Figure 5B).

Sequences from Oo also allow us to systematically characterize transcripts expressed specifically in this stage (Additional file 6). Oo is a transient stage in mosquito, and it has been difficult to obtain sufficient parasite material for genome-wide analysis. Although the majority of the sequences we obtained were transcripts from mosquito, the sequences allowed us to identify many genes that were upregulated and uniquely expressed at this stage, including genes encoding Oo capsule protein and Oo secreted-proteins. On the other hand, there were also 26 genes that encode conserved *Plasmodium *proteins and are specifically expressed or upregulated in Oo. Our work represents the first large-scale transcriptional analysis from the Oo stage of *P. falciparum*. The information presented here provides new insights into gene expression and regulation in this transient stage.

## Conclusions

Although the majority of the gene models in the *P. falciparum *genome have been correctly predicted and/or verified, it is still necessary to continue to sequence cDNA from different developmental stages, particularly those of non-erythrocytic stages. Gene expression studies should be based on directional cDNA libraries, because the presence of antisense transcripts may lead to erroneous conclusions on gene expression and regulation. The presence of antisense transcripts in some gametocyte and ookinete genes suggests that these antisense RNAs may play an important role in gene expression regulation and development of these stages.

## Methods

### Parasite cultures and synchronization

*P. falciparum *strain 3D7 was cultured as previously described [44]. Briefly, malaria parasites were cultured in T_150 _flasks with RPMI 1640 (KD Medical, Columbia, MD, USA) supplemented with 10 μg/ml gentamicin (Gibco, Gaithersburg, MD, USA), 0.25% NaHCO_3 _(Gibco) and 0.5% albumax (Invitrogen, Carlsbad, CA, USA). Schizonts (Sc) were enriched and collected using a Percoll-sorbitol gradient 60-40% (Amersham Biosciences, Piscataway, NJ, USA) and Sigma, St. Louis, MO, USA, respectively), washed with pre-warmed incomplete medium twice, and further cultured under standard culture conditions (37°C in 90% nitrogen, 5% CO_2 _, and 5% O_2_) for 6 h. Following re-invasion, parasites were further synchronized as described [44]. Briefly, parasites were treated with 5% sorbitol at 37°C for 15 min, washed with pre-warmed incomplete medium twice, and placed back in culture. After culture for one full cycle, samples were harvested at 8, 19, 30, and 42 h post infection (hpi) corresponding to R, ET, LT, and Sc stages, respectively.

Gametocytes were produced from asexual cultures of 3D7 by the method of Ifediba and Vanderberg [45]. Briefly, the culture was set up in a T_150 _flask at 0.2% parasitemia and 6% hematocrit in 25 ml of complete medium supplemented with 10% human serum. The culture was maintained under standard culture conditions, fed 25 ml medium on days 2 and 3, and then fed daily with 50 ml medium from day 4 until harvest. On days 6-8, 50 mM N-acetyl-glucosamine (NAG) and 60 nM pyrimethamine were included in the medium to eliminate asexual parasites. Stage II gametocytes (GII) were harvested on day 8, and GV were harvested on day 15. Parasites were isolated by centrifugation, washed in PBS, and incubated in 0.015% saponin (Sigma) in PBS for 10 min at room temperature. The parasites were recovered by centrifugation, and saponin treatment was repeated 1 or 2 times until all red blood cells were removed. The parasites were washed in PBS and the cell pellet stored at -80°C until use.

Oo were harvested from 30 mosquito midguts 24 h after a *P. falciparum *infected blood meal. Briefly, *An. gambiae *strain L35 5-day-old females were fed with 3D7 cultures of GV at a parasitemia of 0.5-1%. G cultures were diluted four times with fresh blood prior to mosquito feeding. A subset of 19 mosquitoes was left for assessing midgut infection at day 8 post feeding. Microscopic observation of mercurochrome-stained midguts revealed a mean value of 52 Oo per midgut.

### cDNA libraries preparation

Total RNA was isolated using TRIZOL (Invitrogen), precipitated with isopropanol, and washed with 70% ethanol. Dynabeads^® ^mRNA purification kit (Invitrogen) was used to purify polyA^+ ^RNA from 10 μg of total RNA. First-strand cDNA was obtained using Superscript III First cDNA Strand (Invitrogen) following manufacturer's instructions. Second-strand cDNA was obtained using *Escherichia coli *polymerase I (Invitrogen). Double-stranded cDNA was purified using QIAquick PCR purification kit (Qiagen, Chatsworth, CA, USA). Purified cDNA (350 ng) in 40 μl TE buffer was processed in an ice-cold Bioruptor at medium power for 30 min to obtain DNA fragments of 200-500 bp. Fragmented cDNA was blunt-ended using End-It repair kit (Epicentre Biotechnology, Madison, WI, USA) and further purified with QIAquick PCR purification kit (Qiagen). Addition of polyA was performed by incubating the cDNA fragments at 70°C in the presence of Taq DNA polymerase (New England BioLabs, Beverly, MA, USA) and dATP 1 mM for 30 min. Purified cDNA fragments were ligated to 1:10 diluted Illumina adaptor oligo mix using T4 DNA ligase (New England BioLabs). Ligated DNA was PCR amplified using finnzymes high-fidelity DNA polymerase master mix (New England BioLabs) and the PCR primers PE 1.0 and 2.0 (Illumina Inc., San Diego, CA, USA). PCR products were again purified and sequenced using the Illumina 2 G genome analyzer.

### Strand-specific RNA-seq libraries preparation

Strand-specific RNA-seq libraries were constructed according to the method described by Parkhomchuk [46]. Briefly, 300 ng of polyA^+ ^RNA was used to generate the first strand of cDNA as described above for regular Illumina libraries. Before second-strand synthesis, the sample was purified using 350 μl Sephadex G-50 columns (GE Healthcare, Piscataway, NJ, USA) to remove traces of dNTPs. The second strand of cDNA was synthesized using a second-strand synthesis kit (Invitrogen) replacing dTTP with dUTP (Applied Biosystems, Fullerton, CA, USA). Double-stranded cDNA was fragmented as described above and ligated to indexed-Illumina adapters. Prior to library amplification by PCR, the cDNA was digested with 1 U of uracil-n-glycosylase (Applied Biosystems) at 37°C for 15 min.

### Illumina sequencing

Regular bidirectional libraries were sequenced after 36 cycles of amplification using an Illumina 1 G genome analyzer according to manufacturer's instructions. Strand-specific libraries were analyzed in an Illumina 2 G genome analyzer using 51 cycles of amplification and paired-end conditions. In both cases, each sample of amplified material was loaded at a concentration of 4 pM per flow-cell. The four strand-specific libraries were bar coded and loaded together in one single flow-cell.

### Sequence reads alignment and data analysis

Short reads (36 bp or 51 bp reads) obtained were processed using Illumina Pipeline Analysis. Demultiplexing and removal of 6 bp barcode in 51-bp reads was done with Novobarcode software (Novocraft Technologies, Selangor, Malaysia). Reads were mapped to the *P. falciparum *genome sequence (PlasmoDB v7.1) using TopHat [21]. The parameters were adjusted to allow report of a junction if there were at least 7 bp of reads present at each side of the "anchor," with a maximum intron size of 800 bp. For higher accuracy, we filtered the TopHat output to retain only reads that were mapped with 0 mismatches along the entire 36-bp read, or up to 1 mismatches in longer reads. Subsequent filtering of junctions with only one spliced read and/or overlapping repeat regions was done with custom shell scripts as well as BEDTools v. 2.6 [47]. Repeat regions were determined using the positions of reads that Bowtie could map to more than one genomic region. Differential expression of genes was calculated using Cufflinks [48], and the fold change of the fragments per kilobase of exon per million [FPKM] fragments mapped value between each stage and R stage was reported after a cutoff of 5 was applied.

The strand-specific libraries were analyzed essentially the same way as the bidirectional libraries with the exception of allowing one mismatch at each segment of the reads (fragment of read that is mapped independently when searching for junctions. The minimum fragment size was established as 22 bp (approximately half the length of the 46-bp reads remaining after demultiplexing), and the anchor was 10 bp with no mismatches allowed.

Exons with substantial amounts of antisense were identified on the basis of having > 70% of reads mapping in the antisense direction and having at least 150 reads. This simple thresholding method identifies genes with compelling evidence for antisense but may miss some transcripts and is arbitrary. Because many exons had low numbers of mapped reads, using the ratios of sense transcription as a quantitative measure of antisense would be plagued by noise. Furthermore, a cutoff based on the total number of antisense reads would yield false positives for highly transcribed genes, because the digestion of the second-strand is imperfect. The sequences have been deposited in GanBank with accession number SRP009370 and in PlasmoDB http://plasmodb.org/plasmo/.

### RT-PCR confirmation of intron splicing sites

Reverse-transcriptase-PCR reactions were performed with RNA samples from the same time points of the parasite life cycle used to prepare the libraries. cDNA was obtained from 2 μg of total RNA using QuantiTect^® ^reverse transcription (Qiagen) following manufacturer's recommendations. PCR reactions were performed using 200 ng of the cDNA and gene-specific primers (Additional file 7).

## Abbreviations

bp: base pairs; cDNA: complementary DNA; DOZI: development of zygote inhibited; EST: expressed sequence tag; ET: early trophozoite stage; GII: gametocyte stage II; GV: gametocyte stage V; hpi: hours post invasion; LT: late trophozoite stage; NAG: N-acetyl-glucosamine; NAT: natural antisense transcript; Oo: ookinete; PCR: polymerase chain reaction; RPKM: reads per kilobase per million reads sequenced; R: ring stage; Sc: schizont; U: enzymatic unit; UTR: untranslated region.

## Authors' contributions

MJL-B performed carried out experiments, data analysis, and writing; JL and MQ performed data analysis and writing; KW was responsible for gametocyte materials; AM-C and CB-M mosquito infections with gametocyte cultures, and Oo Illumina sequences done by NISC Comparative Sequencing Program; KC and KZ carried out sequencing and analysis; X-z.S performed data analysis and writing. All authors read and approved the final manuscript.

## Supplementary Material

Additional file 1 **Novel junctions discovered after analysis of the bidirectional RNA-Seq libraries from seven time points of *Plasmodium falciparum *life cycle**. Click here for file

Additional file 2 **Exons with high levels of antisense (-log *P *> 150) transcripts from the strand-specific libraries**. Click here for file

Additional file 3 **Functional enrichment of genes with high levels of antisense transcripts**. Click here for file

Additional file 4 **Splicing junctions detected using 1 or 10 bridging reads, respectively**. Click here for file

Additional file 5 **New intro-exon junctions detected using reads from the strand-specific libraries and two previous studies**. Click here for file

Additional file 6 **Genes expressed at levels significantly different from ring-stage (fold changes)**. Click here for file

Additional file 7 **Validation of splicing junctions and alternatively spliced events detected**. Click here for file
